# Mucocèle ethmoïdale post-radique

**DOI:** 10.11604/pamj.2015.22.222.8119

**Published:** 2015-11-10

**Authors:** Rim Lahiani, Madiha Mahfoudhi

**Affiliations:** 1 Service ORL, Hôpital Charles Nicolle, Tunis, Tunisie; 2 Service de Médecine Interne A, Hôpital Charles Nicolle, Tunis, Tunisie

**Keywords:** Mucocèle, sinus ethmoïdal, radiothérapie, mucocele, ethmoid sinus, radiotherapy

## Image en medicine

Les mucocèles sont des complications tardives et rares de la radiothérapie. Ils sont le plus souvent de siège ethmoïdal postérieur ou sphénoïdal. Ils peuvent exceptionnellement entrainer des troubles visuels graves par compression du nerf optique. Le diagnostic est évoqué par les données cliniques et de l'imagerie. Le traitement est chirurgical avec un examen histologique systématique afin d’éliminer une récidive tumorale. Patient âgé de 48 ans avait des antécédents d'un cancer du cavum (UCNT: undifferenciated carcinoma of nasopharyngeal type) classé T2 N1 M0, traité par chimio et radiothérapie, 3 ans auparavant, avec évolution favorable. Il a été hospitalisé pour épistaxis gauche de faible abondance et douleur orbitaire homolatérale évoluant depuis 3 mois. L'examen physique a objectivé un cavum libre, un état général conservé et des aires ganglionnaires libres. La TDM du massif facial a révélé une formation éthmoïdale gauche, ovoïde, de 3 cm de grand axe, qui amincit les parois osseuses et bombe dans le secteur postéro-interne de l'orbite avec un cavum libre. L'IRM du massif facial a trouvé une lésion kystique de 3 cm de grand axe en hypersignal T1 et hypersignal T2, de siège éthmoïdal postérieur, s’étendant au niveau de la paroi interne du massif orbitaire gauche et comprimant le nerf optique homolatéral. Une récidive du cancer du cavum a été suspectée. Le patient a bénéficié d'une marsupialisation par voie endoscopique. L'examen anatomo-pathologique était en faveur d'une mucocèle. L’évolution était favorable avec un recul d'un an.

**Figure 1:**
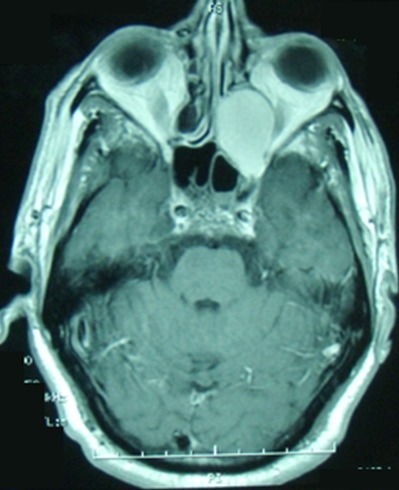
IRM du massif facial (coupes axiales, en séquence T1): Lésion kystique de 3 cm de grand axe en hypersignal T1 de siège éthmoïdal postérieur gauche, comprimant le nerf optique homolatéral

